# Ligation of the Femoral Artery in a Case of Femoro-Popliteal Aneurism

**Published:** 1887-02

**Authors:** J. McF. Gaston

**Affiliations:** Professor of Surgery


### LIGATION OF THE FEMORAL ARTERY IN A CASE OF FEMORO-POPLITEAL ANEURISM.

BY J. MCF. GASTON, M. D., PROFESSOR OF SURGERY.

**A Clinical Lecture delivered January 5, 1887, at the Southern Medical College, Atlanta, Georgia.**

*Reported for the Southern Medical Record.*

GENTLEMEN : I will occupy a portion of the hour allotted to the clinic to-day with some observations upon the case in which the femoral artery was ligated on the 26th of November, 1886, for the obliteration of a popliteal aneurism, while there was a femoral aneurism on the cardiac side of the ligature, which was left for another operation. This patient was referred to me by Dr. S. W. Stiles. After having used mechanical means of pressure over the pubic bone for a month without securing any satisfactory result, I availed myself of the assistance of volunteers from the Class, in details of four to each four hours, to keep up manual compression upon the femoral during three days and nights, and still the pulsation continued in both aneurisms. There had been at one period indications of thickening of the dilated sac in the popliteal space, but upon suspending the means of compression the deposition ceased entirely. Most of the Class had an opportunity of examining both of these tumors by sight, touch and hearing, and will doubtless recognize the conformation, the bounding impulse, and the peculiar whirr of an aneurism as quite distinct from other protuberances. You are all aware that there are spontaneous and traumatic aneurisms, the former depending upon degeneration and dilatation of the arterial coats from various causes.

This man, about thirty years old, has enjoyed good health in his occupation as a shoemaker, until recently, when he first felt some inconvenience in the ankle of the left leg ; then a protuberance was felt beneath the knee, and finally the enlargement appeared some three inches below the pubic bone. It was upon the thigh of this leg that he was accustomed to hammer his leather and peg the shoes, held by a strap passing around the knee and beneath the foot, so that we may perhaps look to this constraint of the limb, and the consequent impediment to the free circulation of blood in the artery, as explanatory of the double anuerism, one being above and the other below the point of constriction from the strap. It was found, however, that this condition was associated with a slight bellows sound of the heart and the extension of the systolic impulse along the aorta without, however, any well defined valvular trouble. This cardiac derangement had become augmented under the worrying ordeal of the protracted compression, so that it was not thought proper to continue it. Under such circumstances, and in accordance with the views of my colleague, Dr. W. D. Bizzell, it was determined to operate without further delay ; and you will recollect that in my remarks previously the reasons were given for not proceeding to a radical operation by ligature above the femoral aneurism : first, that the shutting off the blood supply through the profunda would endanger the proper nourishment of the limb ; secondly, if the ligature was effected below Poupart's ligament the structural integrity of the coats of the vessel in such proximity to the aneurismal dilatation might not be sufficient for safe ligation, and, thirdly, if the external iliac should be selected for ligation, the neighboring bifurcation of the internal iliac would project the current of blood into the arterial trunk, so as to interfere with the solidification of the clot above the ligature. It was considered likewise that the ligation upon the distal side of the femoral aneurism might lead to its obliteration, as the dilatation was, perhaps, below the departure of the profunda. In view of these facts, I concluded, with the advice of my colleagues, to ligate the femoral below Scarpa's triangle, just where it enters Hunter's canal. By an incision of three inches through the integument, below the sartorious and between the rectus femoris and the adductor magnus, the femoral was reached, and passing a ligature of Czerny silk with the aneurism needle, a knot was tied around it, so as to completely obstruct the flow of blood. There was cessation of pulsation in the popliteal space below, and cutting short the ligature I closed the wound by interrupted suture. Arrest of the flow of blood in the aneurism was verified by several colleagues on the occasion, and there was no subsequent return of the circulation to it. A number of the Class were so kind as to watch the case after recovering from the effects of the A. C. E. anæsthetic, and to apply bottles of warm water to the foot and leg during the night, so that no considerable diminution of temperature occurred in the limb, nor was there any marked constitutional disturbance or vital depression. The general indications by the thermometer were slightly below normal for a few days, and afterwards some elevation of temperature, accompanied by turgescence of the venous circulation and tumefaction of the parts around the aneurismal induration in the popliteal region. A roller bandage, applied from the foot around the leg and thigh, with frictions of spirits of turpentine and camphor, relieved the swelling and contributed to the comfort of the patient. Within a week after the operation it was apparent that pus had formed in the wound and a few of the stitches of Snowden's iron dyed silk were removed from the central point of the incision, and a free discharge occurred for two weeks, but there was no appearance of the ligature with the pus, and the wound is now closed, leaving some induration near it.

After the expiration of a week it was observed that three large serous collections had formed beneath the thickened epidermis of the sole of the foot, and at the end of two weeks another appeared on the outer side of the foot, containing sanguinolent fluid, which resulted doubtless from impaired capillary circulation. The aneurismal tumor became quite solid after the ligation and imparted a sense of resistance upon grasping it, which continued for ten days.

The hardness has been gradually disappearing since, and there is at present but little induration in the mass, which has diminished from the size of a goose egg to that of a hen's egg, without any abnormal sensibility upon being handled.

It is inferred that the vis a tergo of the heart's impulse being removed by occluding the femoral artery, the blood which distended the dilated pouch formed by the relaxed coats of the popliteal artery remained in this sac to undergo coagulation, and subsequently became absorbed or vascularized. The slow, but constant, diminution of the mass indicates that it will be eventually obliterated and that no portion of the blood will flow through this portion of the vessel, but that the circulation has been established through the anastimosing vessels, communicating in part with the ramifications of the internal iliac. For a time the force of the impulse in the femoral aneurism was lessened, but now it has increased with some augmentation of the size of the protuberance, which is very perceptible to the eye. The sound, upon placing the ear in contact with the tumor, is characteristic of an aneurism, having the bruit well defined, corresponding to the entrance of the blood into the sac from the opening of the artery. It is clearly a sacculated aneurism with lateral projections, so that the largest diameter lies transversely to the femoral artery, the trunk of which presents no perceptible enlargement above the upper border of the tumor. When my thumb is placed firmly over the artery upon the pubic bone all pulsation ceases in the sac and there is a perceptible diminution in its size.

Having waited for the collateral circulation to be fully established, and for the restoration of the lost skin upon the bottom of the foot, which was doubtless due to deficient peripheral circulation, the general condition of the patient has also improved, and it is expected that the ligature of the pubic division of the artery may be undertaken soon with a view to the obliteration of the existing femoral aneurism.

The hope of a cure from the distal ligation has not been realized, and though it was supposed that a partial stenosis occurred in the artery from the continuous pressure over the bone for such a length of time, inducing inflammation of its coats, it has not been attended with any diminution of the aneurism. It is supposed that the thickening of the walls of the vessel may favor its occlusion when it is ligated ; so that a good result is anticipated from the operation near the pubic bone now, though it was not thought advisable previously.

NOTE.—This record is made when going to press (January 25, 1887). There have been indications of cardiac and aortic trouble, with nervous disturbance, amounting, at times, to mental perturbation, which lead to the inference that the arch of the aorta may be implicated. The bellows sound has assumed more of a whizzing character, audible in all parts of the thorax, and evidently extending along the course of the artery. There is not, however, any marked impulse against the thoracic wall, such as would result from any considerable aneurismal dilatation The femoral aneurism has increased notably since the report of the Clinic, and its pressure upon the adjacent nerves is causing pains about the hip and surrounding parts, with aggravation of the suffering when pressure is made over the pubic bone for temporary interruption of the flow of blood. Manipulation of the aneurismal dilatation, when the circulation is cut off, indicates thickening of the walls and is painful. Notwithstanding the unfavorable surroundings, ligation seems to be the choice of evils at present, for relief to the sufferings from the femoral aneurism.

